# Bacteriology

**Published:** 1890-02

**Authors:** 


BACTERIOLOGY.—The bacterial origin of malaria was suspected many years ago, even before the invention of the microscope. In a treatise by Marcus Terentius Varro, *De Re Rustica*, written about 114 B. C., the following passage occurs in a chapter giving directions for the building of a house in the country: "Attention must be also paid as to whether there are marshy places, but for the same reasons, and because when they become dry, certain minute animals breed, which the eye can not discern, and which borne through the air, penetrate into and within the body, by the mouth and nostrils, and propagate obstinate diseases. A situation on which the sun shines all day is more salubrious, for if any animalcules breed and are brought there, they are either blown away or they soon perish from drought." The passage is certainly a remarkable one, although it is impossible that Varro could have possessed any of our modern knowledge on the subject. —*Popular Science News*, December, 1889.

THE French law gives a physician's claim against the estate of a deceased patient precedence.

